# Preparation TiO_2_ core-shell nanospheres and application as efficiency drug detection sensor

**DOI:** 10.1186/1556-276X-9-465

**Published:** 2014-09-03

**Authors:** Jingli Yue, Zhenhua Chen, Yifeng E, Lianshan Chen, Jing Zhang, Yimeng Song, Yuchun Zhai

**Affiliations:** 1 School of Material and Metallurgy, Northeastern University, 110004 Shenyang, People’s Republic of China; 2 College of Pharmacy, Liaoning Medical University, 121001 Jinzhou, People’s Republic of China; 3 College of Chemistry, Nankai University, 300071 Tianjin, People’s Republic of China

**Keywords:** Titanium dioxide, Nanoparticles, Sensors, Diltiazem hydrochloride

## Abstract

In this paper, we report the facile preparation of monodisperse titanium dioxide-diltiazem/tetrachlorobismuth core-shell nanospheres (TiO_2_@DTMBi), in which, diltiazem (DTM)/tetrachlorobismuth (BiCl_4_) complexes were employed as electroactive materials. The morphology, size, formation, and structure of the obtained TiO_2_@DTMBi spheres were investigated by transmission electron microscopy, scanning electron microscopy, dynamic light scattering, Fourier transform infrared spectroscopy, and X-ray diffraction. The optimal condition of obtained monodisperse 40-nm TiO_2_@DTMBi spheres was researched. The results of using TiO_2_@DTMBi nanospheres as proposed drug sensor indicate a wide linear range (10^-7^ to 10^-1^ M) and a very low detection limit of 0.20 μg/mL.

## Background

Monodisperse nanoparticles have continued to arouse interests due to their broad range of applications in biological and biomedical applications, such as drug and gene delivery vectors, bioimaging agents, chemical, and biological sensors [1-5]. The sensing of biological agents, diseases, toxic materials, and drugs is always an important goal for biomedical diagnosis and forensic analysis [4]. Because the attachment of metallic and semiconductor nanoparticles onto electrodes drastically enhances the conductivity and electron transfer from the redox analytes, these nanoparticles have been widely applied to electroanalytical sensing [6]. Among various metal oxide nanoparticles, owing to its low cost, good electrochemical performance, and hydrophilic surface, TiO_2_ nanoparticles have been typically and widely used in biological and electroanalytical sensing fields, especially in aqueous systems [4,5]. Thus, fabrication of monodisperse TiO_2_ nanoparticles have always attracted much attention [5,7-9]. However, so far there is lack of knowledge regarding using TiO_2_ nanoparticles as drug detection sensor. Here in, the present work aims to investigate TiO_2_ nanospheres as high-efficiency sensor for detection of diltiazem, a drug commonly used in the treatment of hypertension, angina pectoris, and some types of arrhythmia.

Recently, a few investigations focused on potentiometric membrane as sensors used for the analysis of different kinds of drugs including of diltiazem: the detection concentration range is approximately 10^-5^ to 10^-1^ M, and the detection limit was about several micrograms per milliliter [10,11]. Though the carbon nanotubes were introduced into the research [11], it seemed to widen the detection concentration range and lowering the detection limit is still a big challenge. By the virtue of TiO_2_ in sensing field [5-7], in the present work, we intend to prepare a sensor with wider linear range and lower detection limit as sub micrograms per milliliter.

## Methods

### Preparation of TiO_2_ nanoparticles (TiO_2_ NPs)

The synthesis of TiO_2_ nanoparticles follows the titanium (IV) butoxide Ti (OC_4_H_9_) hydrolysis method reported before with some modification [7,12]. Briefly, Ti (OC_4_H_9_) (97%, Sigma-Aldrich, St. Loius, MO, USA) was dissolved in distilled water at room temperature to form an aqueous solution of 0.12 mol/L. After stirring for 12 h, the prepared solution was kept in a water bath under approximately 80°C without stirring for 3 h. The obtained white precipitates were alternately rinsed by distilled water and ethanol thoroughly, then, they were ultrafiltered through 0.22-μm pore-size filters to remove the insoluble impurities. Finally, after centrifugally separated from solution, the fabricated nanoparticles were dried at 120°C for 20 h and sintered at 600°C for 4 h for further characterization and application.

### Preparation of TiO2@DTMBi core-shell nanospheres

**Figure 1:**
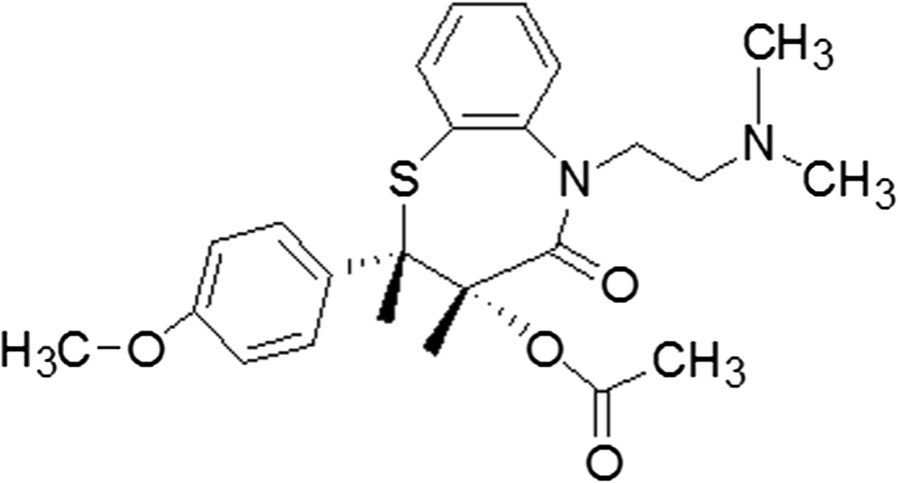
Chemical structure of diltiazem hydrochloride.

In a typical procedure (T1 system, Table 1), 0.01 mol TiO_2_ NPs were added into a 50.0-mL solution which contain 0.01 mol Bi (NO_3_)_3_ · 5H_2_O (98%, Sigma-Aldrich, St. Loius, MO, USA) and 0.1 mol HCl to form a mixture under ultrasound conditions. Subsequently, the mixture was added into a 50.0-mL, 0.01-mol/L diltiazem hydrochloride (Fluka, structure shown in Figure 1) solution drop by drop under vigorous stirring. The resulted precipitates were thoroughly rinsed by distilled water and ethanol alternately. After dried at 60°C for 10 h, the products were collected for further characterization and application. The other systems follow the same steps with different molar ratio of DTMBi/TiO_2_ as listed in Table 1.

**Table 1:** **Key parameters of obtained TiO**_**2**_**@DTMBi NSs and drug detection results**

**Sample**	**DTMBi/TiO**_**2 **_**(molar ratio)**	**Morphology**	**Detection limit (μg/mL)**
T0	No TiO_2_	Aggregates	1.53
T1	1:1	Core-shell spheres	0.20
T2	2:1	Aggregates	1.12
T3	1:2	Aggregates	0.94

### Preparation of TiO_2_@DTMBi nanospheres modified membrane electrodes

According to the literature [10], the general procedure to prepare TiO_2_@DTMBi nanospheres (NSs) modified polyvinylchloride (PVC) membrane was as follows: 5.0-mg TiO_2_@DTMBi NSs along with 30.0-mg PVC, and 65.0-mg dibutyl phthalate (DBP) were dispersed in 5.0-mL tetrahydrofuran (THF) to form a mixture. The resulting mixture was transferred into a glass dish. The solvent was evaporated slowly until an oily concentrated mixture was obtained. A Pyrex tube (4 mm o.d.) was dipped into the mixture for approximately 8 s so that a transparent membrane of about 0.3-mm thickness is formed. The tube was then filled with 1.0-mM DTM solution and soaked in 1.0-mM DTM solution for 24 h before used as membrane electrode.

### Preparation of standard diltiazem hydrochloride solutions

A stock solution of 0.1 M diltiazem hydrochloride was prepared. The working solutions (10^-7^ to 10^-1^ M) were prepared by serial appropriate dilution of the stock solution.

### Characterization

To identify the composition of the synthetic products, Fourier transform infrared spectroscopy (FTIR) was performed by using a SHIMADZU spectrum system (SHIMADZU, Kyoto, Japan) with a resolution of 4.00 cm^-1^. The structure of the products was characterized by X-ray diffraction (XRD) using a SHIMADZU X-lab 6000 X-ray powder diffractometer with Cu Kα radiation. The morphologies of the products were studied by scanning electron microscopy (SEM, Hitachi, S4800, Tokyo, Japan) and transmission electron microscopy (TEM, JEM-1200EX, Tokyo, Japan). The mean diameter of the corresponding sample was performed by using dynamic light scattering (DLS, Malvern, Nano ZS90, Worcestershire, UK). The electrochemical data were obtained using a CHI660C electrochemical workstation using cyclic voltammetry and electromotive force measurements. The typical cell for electrochemical data measurement was assembled as follows:

Ag-AgCl | internal solution, 1 mM DTM | PVC membrane electrode | sample solution | Hg-Hg_2_Cl_2_, KCl (satd.).

## Results and discussion

### Morphology of TiO_2_@DTMBi NSs

**Figure 2:**
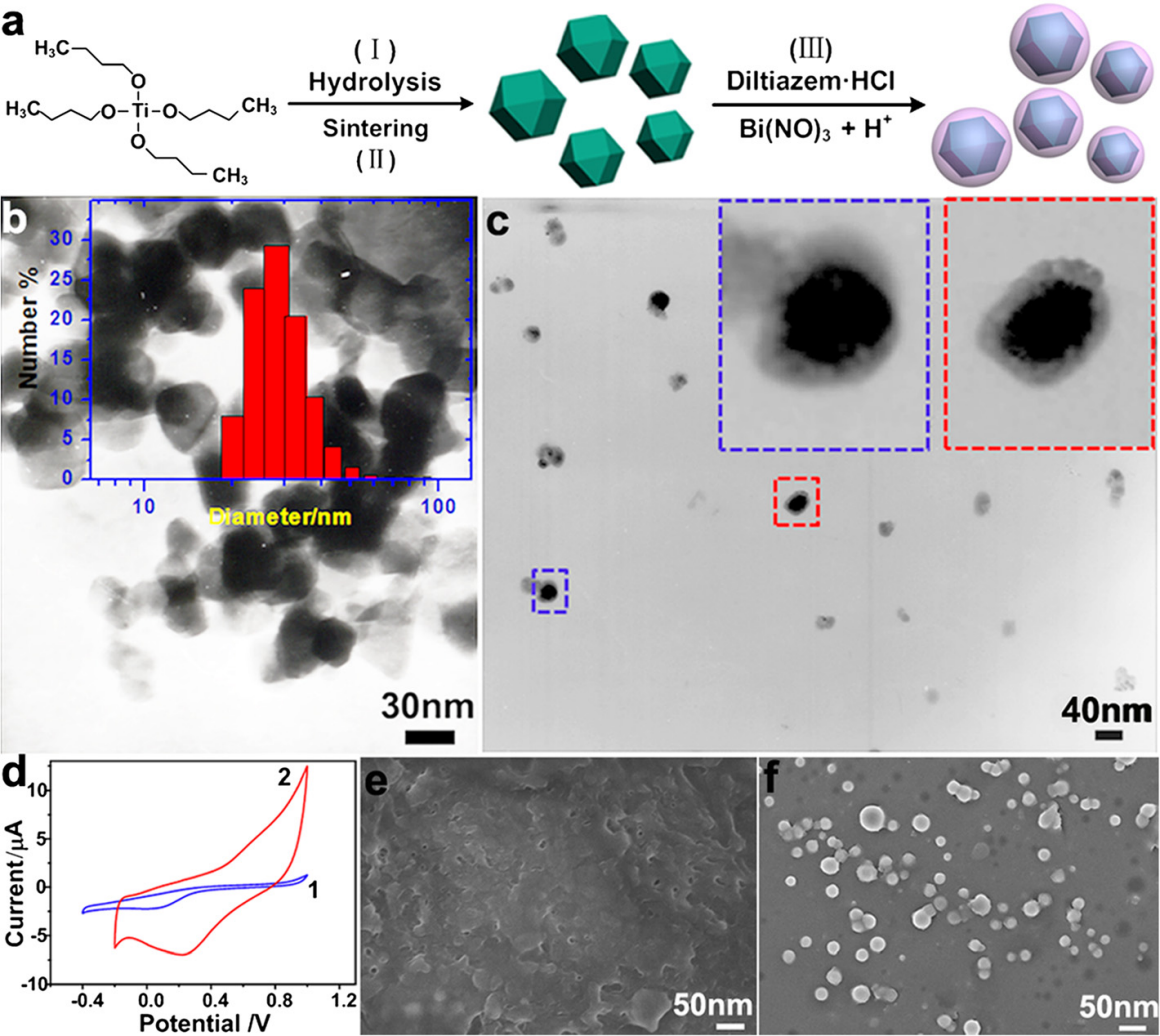
**Schematic illustrations, TEM, cyclic voltammograms, and SEM images. (a)** Schematic illustration of preparation of TiO_2_ nanoparticles and TiO_2_@DTMBi core-shell nanospheres. **(b)** TEM image of TiO_2_ nanoparticles; the insert is size distribution. **(c)** TEM images of TiO_2_@DTMBi core-shell nanospheres; the inserts are two magnified spheres. **(d)** Cyclic voltammograms of electrodes (1), T0 and (2) T1. SEM images of the electrode surface **(e)**, T0 and **(f)** T1.

Figure 2a shows the schematic Ti (OC_4_H_9_) hydrolysis route of preparation of TiO_2_ nanoparticles and TiO_2_@DTMBi core-shell NSs. The TEM image in Figure 2b reveals the obtained TiO_2_ NPs having the size of approximately 30 nm. DLS result (Figure 2b insert) further confirms the average diameter of TiO_2_ NPs that is 31.5 nm. Figure 2c indicates the obtained TiO_2_@DTMBi nanospheres having the size of approximately 40 nm. The magnified TEM images (Figure 2c inserts) show the selected spheres (indicated by the rectangles) having approximately 30 nm TiO_2_ core and approximately 5-nm thickness shell.

### Sensor properties of TiO_2_@DTMBi NSs

**Figure 3:**
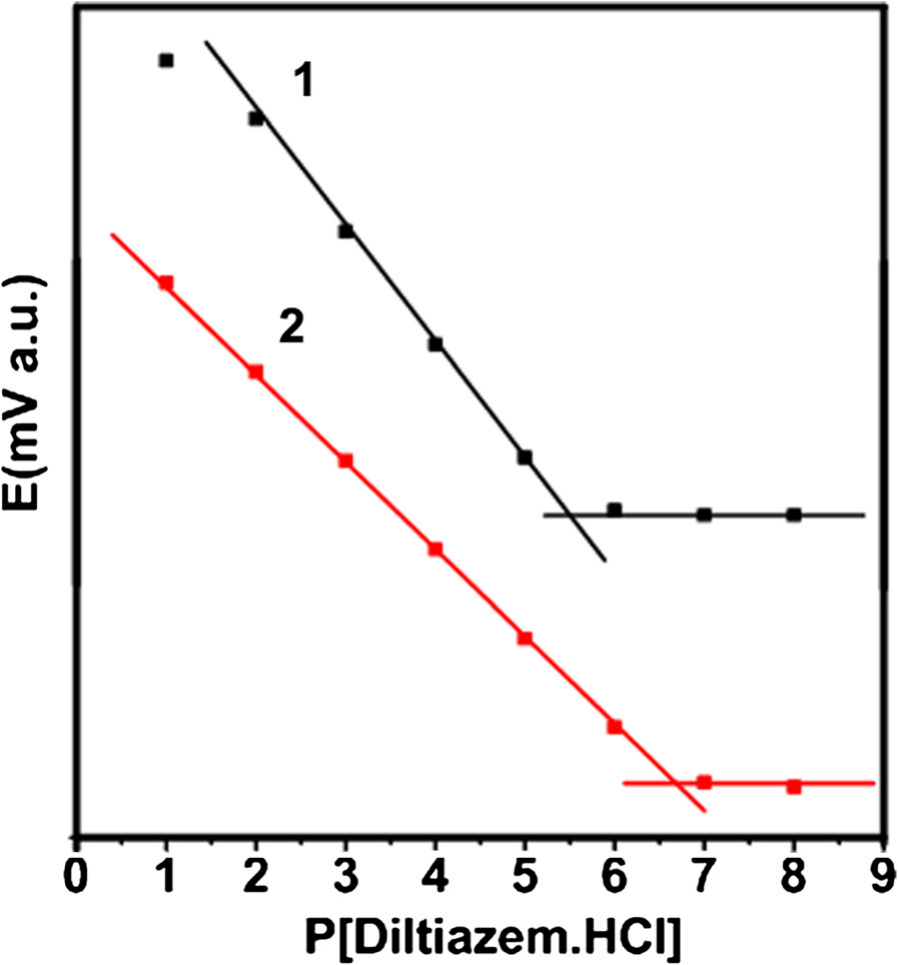
**The calibration curve of using (1) DTMBi and (2) TiO**_**2**_**@DTMBi core-shell nanospheres as detection sensors.**

The cyclic voltammograms in Figure 1d reveal that the electrode modified by TiO_2_@DTMBi NSs exhibits significantly more electron transfer and current compared to the unmodified one. SEM images show the obvious difference between electrode surface with or without TiO_2_@DTMBi NSs modified; the unmodified electrode surface presents the aggregates of DTMBi complexes with uncertain shape (Figure 2e), while for the modified electrode, TiO_2_@DTMBi NSs can be clearly discerned (Figure 2f). It is obvious that these TiO_2_@DTMBi NSs enhance the conductivity and electron transfer of the modified electrode, thus, the enhanced electro transfer would increase the sensitivity to diltiazem. Figure 3 shows the calibration curves of using direct DTMBi and TiO_2_@DTMBi core-shell NSs as detection sensors. By extrapolating the linear parts of the calibration curves, it can be calculated that the detection range and limit for DTMBi sensor (T0 sample) are 10^-1^ to 10^-5^ M and 1.53 μg/mL, respectively. These results are consistent with the reported results that the detection limits for the most selective electrodes sensors are in the range of 10^-5^ to 10^-6^ M [10]. While for TiO_2_@DTMBi core-shell NSs as detection sensor, in which TiO_2_ nanoparticles were introduced, a wider detection range of 10^-1^ to 10^-7^ M and a much lower detection limit of 0.20 μg/mL than the reported results not using TiO_2_ nanoparticles were obtained. These data suggest that TiO_2_@DTMBi core-shell NSs can be used as a proposed high-performance sensor for diltiazem detection.

### Formation, structure, and optimal preparation condition of TiO_2_@DTMBi NSs

**Figure 4:**
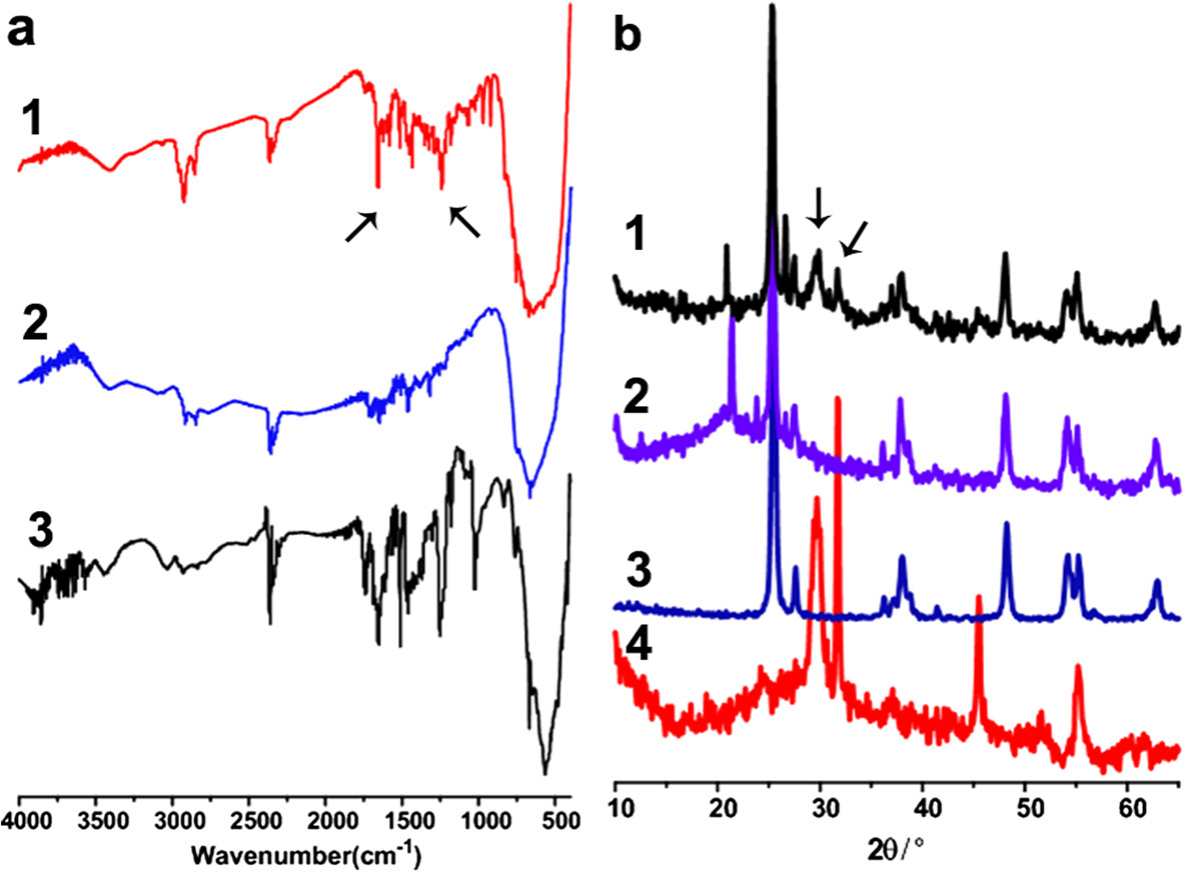
**Infrared spectra and XRD reflection. (a)** Infrared spectra of samples (1) T1, (2) T3, and (3) T0; **(b)** XRD reflection of (1) T1, (2) T3, (3) TiO_2_ NPs, and (4) T0.

FTIR spectra of TiO_2_@DTMBi NSs clearly show the characteristic absorption peaks ascribed to DTM ranging from 1,230 to 1,650 cm^-1^ (Figure 4a (spectrum 1), indicated by the arrows). XRD reflection also shows TiO_2_@DTMBi NSs having the feature peaks of DTM (Figure 4b (spectrum 1), indicated by the arrows). XRD reflections in Figure 4b also indicate that the crystal structure of the obtained TiO_2_ NSs and TiO_2_@DTMBi NSs both mainly belong to anatase titanium dioxide [13], though the small peaks belong to rutile TiO_2_ also been found.

In Figure 4b, XRD peaks of DTM are only visible for T1 sample. This is because T3 sample contains very low content of DTM. This inference is consistent with the FTIR results showed in Figure 4a. FTIR spectrum for T3 sample presents very weak absorption from 1,230 to 1,650 cm^-1^ ascribed to DTM characteristic peaks (Figure 4a (spectrum 2). We deduce that the very low content of DTM in T3 sample was because of the rinsing process. For T1 sample, because the initial ratio of DTMBi/TiO_2_ is much higher than T3 sample, T1 sample contains more amount of DTM after the rinsing process.

**Figure 5:**
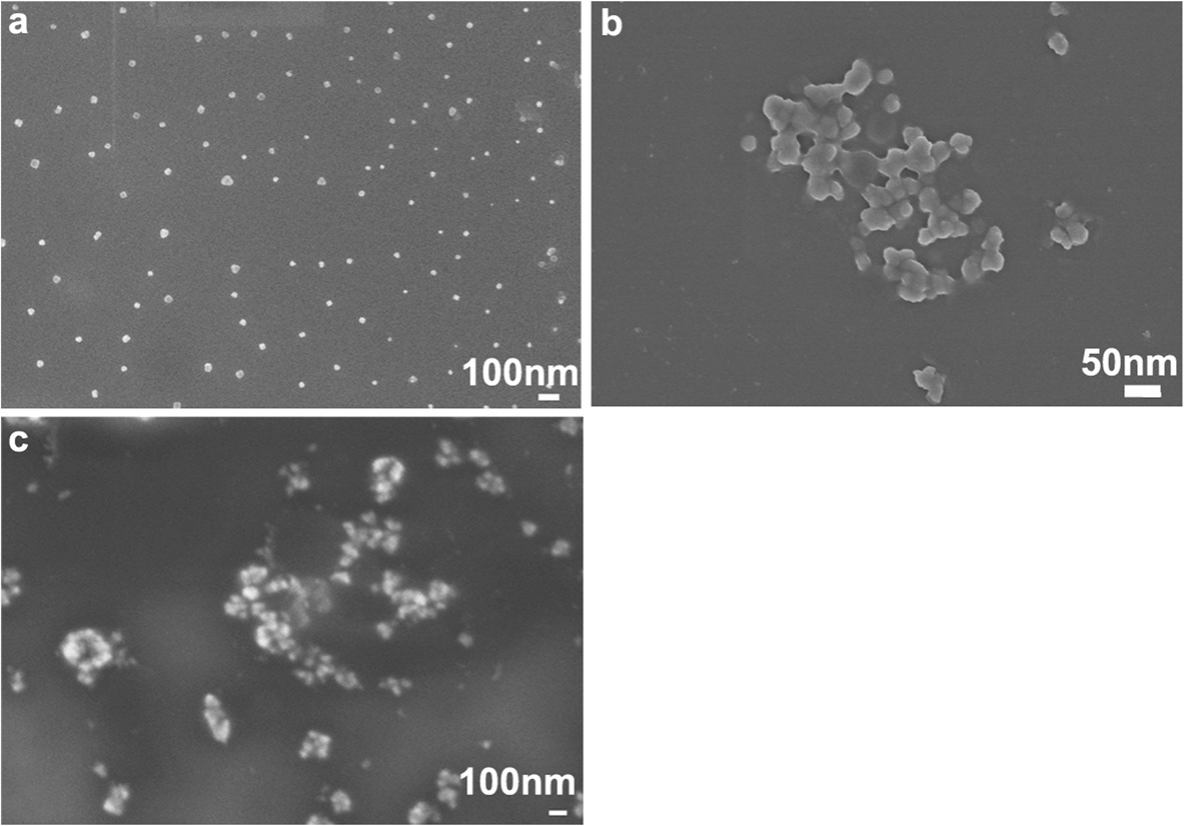
**SEM images of the products obtained under various DTMBi/TiO**_**2 **_**ratio: (a), 1:1; (b), 2:1; and (c), 1:2.**

As illustrated in Figure 2a, there are three preparation steps for TiO_2_@DTMBi NSs, during the third step, it is clear that the DTMBi/TiO_2_ ratio will play an important role in controlling the morphology. We also investigate the effect of different DTMBi/TiO_2_ (molar ratio, listed in Table 1) on the obtained TiO_2_@DTMBi products. As SEM images shown in Figure 5, we can find the monodisperse TiO_2_@DTMBi NSs only been obtained at DTMBi/TiO_2_ = 1:1; the lower or higher ratio both produced much larger aggregates. This might ascribe to the interaction between TiO_2_ and DTM molecules (structure shown in Figure 1) such as hydrogen bond interactions are depended on different DTMBi/TiO_2_ ratio. This inference is according to the literature reports about the H-bond interactions between organic molecules, and crystal particles can modify the growth and assemble of crystal particles [14,15].

### Mechanism for response improvement in the TiO_2_-based system

As far as the mechanism for response improvement in the TiO_2_-based system is concerned, take T1 sample for typical example, we think that evident response improvement is mainly caused by two reasons. One is the response surface area for T1 and T0 (the control) is different. Figure 2e, f reveals that electrode surface for T0 and T1 are totally different; it is obvious that T1 with many nanospheres have bigger response surface area than T0 without TiO_2_ nanoparticles. The other is that those TiO_2_ nanoparticles enhance the conductivity and electron transfer of the modified electrode, thus, the enhanced electro transfer would increase the sensitivity to diltiazem drug. The results listed in Table 1 also indicate that the morphology of the obtained TiO_2_@DTMBi samples play a very important role on the detection limit. T1 sample with monodisperse morphology has a much lower detection limit of 0.20 μg/mL than those of T2 (1.12 μg/mL) and T3 samples (0.94 μg/mL) with aggregate morphology (shown in Figure 5). We deduce that this difference is mainly caused by different response surface area of T1 to T3 samples, monodisperse nanospheres having bigger response surface area than those aggregate ones.

## Conclusions

In summary, monodisperse, core-shell TiO_2_@DTMBi NSs with size of approximately 40 nm were facile prepared. The obtained TiO_2_@DTMBi NSs were also investigated as sensor to detect diltiazem. The results reveal that when these core-shell NSs are used as detection sensor, they can provide a wider detection range of 10^-1^ to 10^-7^ M and much lower detection limit of 0.20 μg/mL than the literature data. These data demonstrate that TiO_2_@DTMBi core-shell NSs can be used as proposed high-performance sensor for diltiazem detection.

## Competing interests

The authors declare that they have no competing interests.

## Authors’ contributions

JY, YE, LC, JZ, and YS took the tasks of experimental, data collection, and draft writing; ZC gave his contributions on the experimental design and guidance, data analysis, as well as the main paper organization; and YZ took the contributions on the research guidance, discussion, and paper modification. All authors read and approved the final manuscript.

## Authors’ information

ZC is a Ph.D. major in Biomedical Engineering, Sichuan University, China. He has focused his research interest on the biomaterials especially on the nanoparticles synthesis and application for more than 7 years. His published papers involved the inorganic and organic nanoparticles toward multifunctional nanocarriers and sensors and biomineralization.
